# Timeliness of Breast Diagnostic Imaging and Biopsy in Practice: 15 Years of Collecting, Comparing, and Defining Quality Breast Cancer Care

**DOI:** 10.1245/s10434-023-13905-6

**Published:** 2023-08-01

**Authors:** Cory Amanda Donovan, Cary S. Kaufman, Kari A. Thomas, Ayfer Kamali Polat, Marguerite Thomas, Bonnie Mack, Ariel Gilbert, Terry Sarantou

**Affiliations:** 1 Surgical Oncology, Legacy Health, Portland, OR USA; 2 Department of Surgery, Bellingham Regional Breast Center, University of Washington, Bellingham, WA USA; 3 Pacific Imaging Associates, Legacy Good Samaritan Breast Health Center, Portland, OR USA; 4 Department of General Surgery, Ondokuz Mayis University, Samsun, Turkey; 5 Oncology Program, Penrose-St Francis Cancer Center, Colorado Springs, CO USA; 6 The Breast Center at Portsmouth Regional Hospital, Portsmouth, NH USA; 7 National Consortium of Breast Centers, Warsaw, IN USA; 8 Levine Cancer Institute, Atrium Health, Charlotte, NC USA

## Abstract

**Background:**

The literature lacks well-established benchmarks for expected time between screening mammogram to diagnostic imaging and then to core needle breast biopsy.

**Methods:**

Timeliness of diagnostic imaging workup was evaluated using aggregate data from 2005 to 2019 submitted to The National Quality Measures for Breast Centers (NQMBC).

**Results:**

A total of 419 breast centers submitted data for 1,805,515 patients on the time from screening mammogram to diagnostic imaging. The overall time was 7 days with 75th, 25th, and 10th percentile values of 5, 10, and 13.5 days, respectively. The average time in business days decreased from 9.1 to 7.1 days (*p *< 0.001) over the study period with the greatest gains in poorest-performing quartiles. Screening centers and centers in the Midwest had significantly shorter time to diagnostic imaging. Time from diagnostic imaging to core needle biopsy was submitted by 406 facilities representing 386,077 patients. The average time was 6 business days, with 75th, 25th, and 10th percentiles of 4, 9, and 13.7 days, respectively. Time to biopsy improved from a mean of 9.0 to 6.3 days (*p *< 0.001) with the most improvement in the poorest-performing quartiles. Screening centers, centers in the Midwest, and centers in metropolitan areas had significantly shorter time to biopsy.

**Conclusions:**

In a robust dataset, the time from screening mammogram to diagnostic imaging and from diagnostic imaging to biopsy decreased from 2005 to 2019. On average, patients could expect to have diagnostic imaging and biopsies within 1 week of abnormal results. Monitoring and comparing performance with reported data may improve quality in breast care.

Timely care is one of the Institute of Medicine identified core quality healthcare values.^1^ Ensuring timely diagnosis and treatment can optimize breast cancer outcomes and decrease patient anxiety.^2–4^ Radiology is the entry point for most patients into a comprehensive breast center.^5,6^ Few large datasets on timeliness of care between screening mammogram and diagnostic imaging and from diagnostic imaging to biopsy have been publicly reported, and fewer still have evaluated trends over time.^7^ It is valuable to know what “timeliness” means for the average patient undergoing breast imaging and what factors influence timeliness of care. Multidisciplinary breast cancer care lacks established benchmarks to define quality care. The Mammography Quality Standards Act (MQSA) requires that mammography facilities conduct an audit of their quality, but does not specify what measures should be included, or identify standards.^8^ National Cancer Database (NCDB) and Commission on Cancer (CoC) collect this data, but reports on quality measures have not highlighted timeliness of diagnostic imaging.^9–11^

National Quality Measures for Breast Centers (NQMBC) is a program for measuring breast cancer care quality developed in 2005 by the National Consortium of Breast Cancers (NCBC). NQMBC is a web-based tool of 42 nationally recognized performance measures that encompass the entire patient journey through a breast center from screening mammogram to treatment and potential side effects. Of the NQMBC measures, timeliness of care has proven to be of particular interest to patients and providers. The NQMBC encourages centers to accurately measure and report the quality of the interdisciplinary care they deliver, focusing on outcomes, not structure. The program allows breast centers to filter the NQMBC database results to provide comparisons with like institutions. The goal is to improve breast cancer care by tracking this data and allowing institutions to compare their data with aggregate results from across the country. The original goal was that institutions could improve quality when their performance was tracked and compared with others (Fig. 1). Initial early results were published in 2010.^12^

**Fig. 1:**
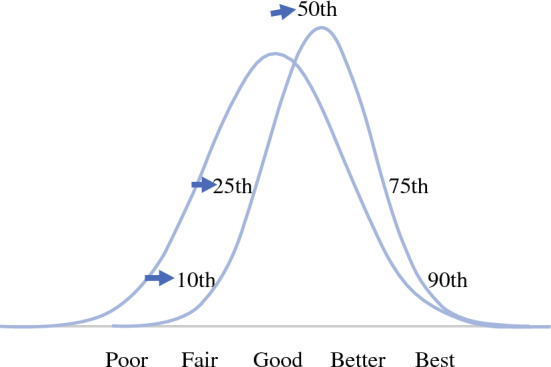
Quality measure: all levels can improve when they compare themselves

NQMBC represents a powerful dataset continuously updated since 2005 for evaluating timeliness metrics at breast centers across the USA and to clarify what factors may influence the timing of diagnostic breast care. Data have been gathered from diverse centers, representing millions of patient visits to develop targets for best practice. We evaluated the NQMBC data for over the past 15 years to look for trends in timeliness of breast cancer and to examine the variables that relate to performance.

## Methods

NQMBC is a voluntary web-based platform. Participating centers have the option to enter the data or not, frequently or not, and enter data for all measures or specific measures they select. Submissions may occur every 6 months (two times per year), and all submissions are averages representing many consecutive patients for the 6-month time period specified. One month of data (30 consecutive patients minimum) is required for each measurement. Several software companies used for mammography reporting have incorporated the NQMBC measures into their software to facilitate data collection. The data provide a representative snapshot of the level of care. To ensure that the submitted data are accurate, a data affidavit is signed by the director of the breast center as well as the data collector. Additionally, mandatory annual deidentified random audits are conducted by NQMBC. Any intentional submission of false data is cause for permanent expulsion from the program. All individual center data are kept confidential by the NQMBC. Centers can instantly compare their results with other centers, which can be sorted to identify similar centers and evaluated over multiple time periods. This program is Health Insurance Portability and Accountability Act (HIPAA) compliant as no individual patient data are ever collected.

All data on time from screening to diagnostic mammogram and from diagnostic mammogram to core needle biopsy was obtained from 2005 to 2019 (the 15 years before the COVID-19 pandemic). Time from screening mammogram to diagnostic mammogram included all patients who had an abnormal screening mammogram for which diagnostic imaging followed, both those that required biopsy and those that did not. Time from diagnostic imaging to biopsy was measured from first diagnostic imaging to first biopsy and included patients who ultimately were found to have benign or malignant pathology. All days were reported as business days, including all days the center was open. Reports were collected for the first 6 months and the last 6 months of each year, for a total of 30 collection periods. Breast center variables included geographic location, center type (screening diagnostic treatment, etc.), NQMBC certification level, population density (metropolitan, urban, suburban, rural), ownership (for/nonprofit, hospital, hospital system), screening mammogram volume and diagnostic mammogram volume, and breast cancer volume.

These data were analyzed by multiple regression analysis of the data and post hoc analysis on categorical variables (Alpine Testing Solutions). Multiple regression was selected as the analysis method because it allows for measurement of a trend over time and a way to assess the relationship between multiple independent variables and a criterion variable. The regression analysis was conducted using the year broken into 6-month intervals (identified as “a” to signify the first 6 months of the year and “b” for the last 6 months of the year) as the predictor and average number of days either between (1) days between screening mammography and diagnostic imaging as the criterion or (2) days between first diagnostic imaging study and first needle/core biopsy as the criterion. Not all institutions provided complete data across all time periods.

## Results

**Fig. 2:**
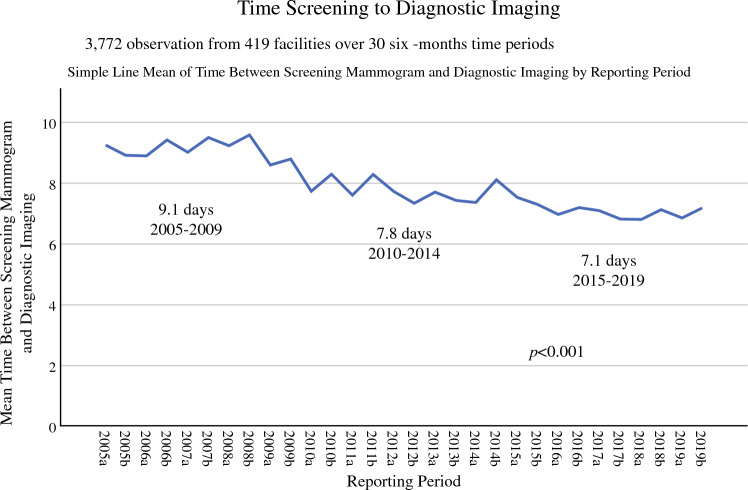
Aggregate data reported to the NQMBC by participating organizations on time from screening to diagnostic mammogram from 2005 to 2019

For the 30 six-month time periods (two per year over the 15 years from 2005 to 2019), the time in business days from screening imaging to diagnostic imaging was available from 419 institutions from 46 states that submitted a total of 3772 observations (aggregate data submissions) representing 1,805,505 patients. Days from screening to diagnostic mammogram overall decreased during the study period from 9.1 to 7.1 days for a decrease of 33% that was statistically significant (regression slope 0.185, *R*^2^ = 0.021, *P* < 0.001) (Fig. 2). All quartiles decreased time to diagnostic mammogram, but the greatest gains were made in the poorest-performing groups (Fig. 2). The average time to diagnostic imaging ranged from 13.5 business days in the lowest 10th percentile to 10, 7, 5, and 3.1 days for the 25th, 50th, 75th, and 90th percentile, respectively.

Variables on univariant analysis associated with shorter time to diagnostic imaging included a screening center, lower diagnostic volume, certified NQMBC breast center of excellence, location in the Midwest or West North Central division, serving a rural population, and categorization as a nonprofit hospital. Variables associated with longer time periods included mammograms performed at a diagnostic center, high diagnostic volume, in the South or Mid Atlantic, in a metropolitan area, and for-profit physician clinics (Table 1). On multivariable analysis, screening centers performed significantly better than diagnostic centers, and centers in the Midwest performed better compared with Southern centers. Timeliness was not associated with screening volume or breast cancer volume.

**Table 1:** Screening mammogram to diagnostic imaging time by variable

Variable	Number of q6-month submissions	Mean date from screening to diagnostic	Std. error
*Geographic division*			
Midwest Region	1097	6.3	0.136
Northeast Region	652	8.3	0.177
West	801	8.4	0.130
South	1210	8.5	0.160
*NQMBC certification*			
Certified quality breast center of excellence	1062	7.127	0.142
Certified participant	508	7.95	0.204
Participant	1774	7.966	0.11
Certified quality breast center	316	8.79	0.258
*Population density*			
Rural	396	6.3	0.229
Suburban	971	7.4	0.147
Urban	1447	7.9	0.120
Metropolitan	958	8.8	0.147
Center type			
Screening	565	6.8	0.193
Clinical	1059	7.5	0.141
Comprehensive	1069	7.8	0.14
Treatment	376	8.5	0.236
Diagnostic	703	8.7	0.173
*Center ownership*			
Nonprofit hospital	741	7.655	0.169
Nonprofit hospital system	2176	7.7	0.099
For-profit hospital system	441	7.8	0.219
Academic or university hospital	214	8.1	0.315
For-profit physician clinic group	195	9.3	0.33
*Patient screening volume quintiles*			
40–60	718	6.68	0.21
60–80	705	6.77	0.21
80–100	711	7.13	0.21
20–40	707	7.46	0.21
Lower 20	696	8.66	0.21

**Fig. 3:**
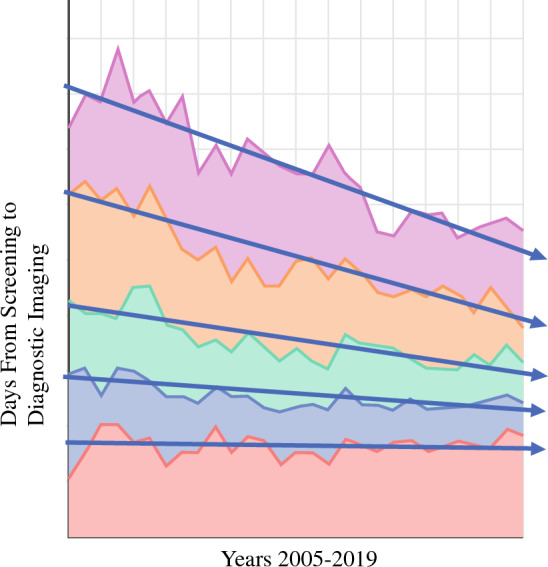
NQMBC aggregate data by quintile from 2005 to 2019 for time from screening to diagnostic imaging by quintile. The quintile reflecting centers with the shortest times to diagnostic imaging is represented in red, while the quintile for centers with the longest times to diagnostic imaging is represented in pink.

**Fig. 4:**
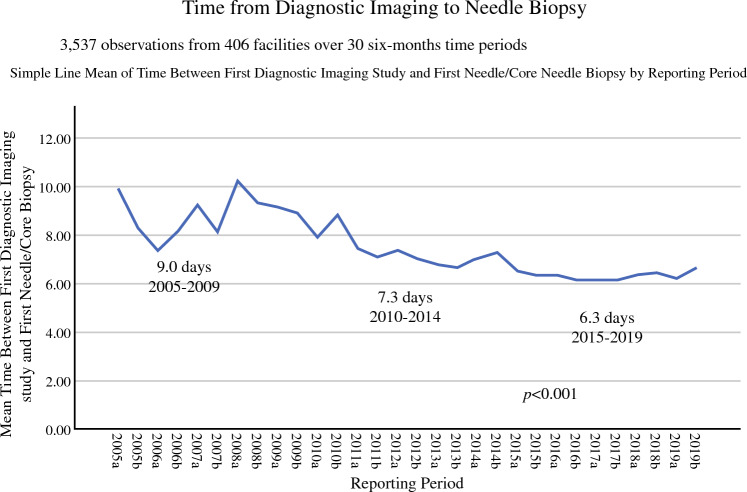
Overall aggregated data from 2005 to 2019 for time from diagnostic mammogram to core needle biopsy

From 2005 to 2019, the time in business days from diagnostic imaging to core needle biopsy was available from 406 facilities from 46 states that submitted 3537 observations capturing data for 356,077 patients. Over the study period, the time from diagnostic imaging to biopsy for the overall group dropped from a mean of 9.0 days for 2005–2009 to 6.3 days for 2015–2019 for a statistically significant overall improvement in 30% over the time period (regression slope 0.250, *R*^2 ^= 0.026, *P *< 0.001) (Fig. 3). Again, this change was most significant for the poorest-performing quartiles (Fig. 4). The lowest 10% performance category or the poorest performers averaged 13.7 business days from diagnostic imaging to biopsy, while the value for the 25th, 50th, 75th, and 90th percentile was 9, 6, 4, and 2 days, respectively.

Factors associated with shorter time periods (better performance) on univariant analysis included screening centers, NQMBC centers of excellence, centers in the West North Central division, metropolitan centers, and nonprofit hospital systems. Variables associated with longer time periods (poorer performance) included treatment centers, NQMBC quality breast centers, the South Atlantic Division, rural centers, and for-profit hospital systems. Elements on multivariable analysis that were statistically significant for shorter time periods include being a screening center, West North Central division, and metropolitan areas. The only variable associated on multivariable analysis with longer time to biopsy was being in the South Atlantic division. Elements that were not associated with time from diagnostic imaging to biopsy were screening volume, diagnostic volume, and breast cancer volume (Table 2).

**Table 2:** Diagnostic imaging to needle biopsy time by categorical variable

Variable	Number of q6-month submissions	Mean date from screening to diagnostic imaging	Std. error
*Geographic division*			
West North Central	208	5.02	0.32
East South Central	183	6.24	0.4
East North Central	703	6.43	0.2
West South Central	313	6.99	0.32
Pacific	529	7.2	0.24
New England	184	7.33	0.4
Mountain	262	8.06	0.33
Middle Atlantic	447	8.08	0.26
*NQMBC certification*			
Certified quality breast center of excellence	1002	6.7	0.18
Participant	1621	7.38	0.14
Certified participant	504	7.68	0.25
Certified quality breast center	308	8.44	0.31
*Population density*			
Metropolitan	900	6.67	0.18
Urban	1386	7.3	0.15
Suburban	945	7.44	0.18
Rural	306	9.1	0.32
*Center type*			
Screening	471	6.44	0.25
Comprehensive	1024	6.86	0.17
Diagnostic	713	7.61	0.21
Treatment	341	7.82	0.3
Clinical	988	7.88	0.18
*Center ownership*			
Non-profit hospital system	2029	7.04	0.12
Academic or university hospital	182	7.12	0.41
For-profit physician clinic group	209	7.24	0.38
Non-profit hospital	698	7.74	0.21
For-profit hospital system	414	8.25	0.27
*Patient diagnostic volume quintiles*			
40–60	715	6.4	0.21
80–100	707	7.24	0.21
20–40	699	7.42	0.21
60–80	712	7.72	0.21
Lower 20	704	7.9	0.21

## Discussion

**Fig. 5:**
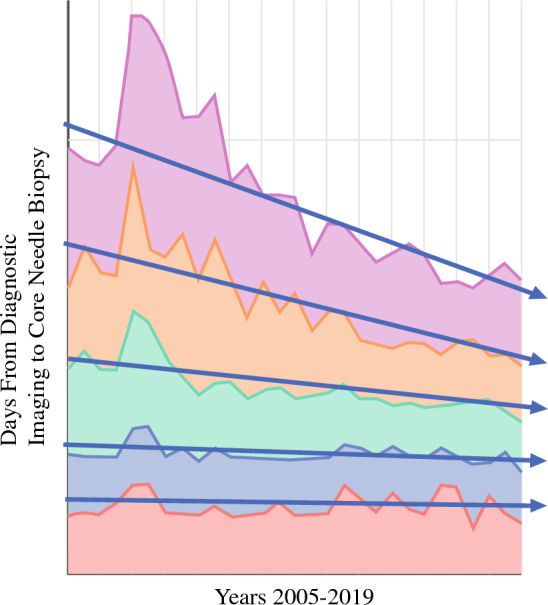
Time from diagnostic imaging to core needle biopsy by quintile. The quintile reflecting centers with the shortest times to biopsy is represented in red, while the quintile for centers with the longest times to biopsy is represented in pink

Using a robust dataset with information from over 400 facilities from 46 states and well over a million patient visits, the NQMBC offers a powerful tool to evaluate trends in timeliness of breast care. Breast centers can and have decreased the time to breast cancer diagnosis. The time from screening imaging to diagnostic imaging and from diagnostic imaging to biopsy decreased consistently and significantly from 2005 to 2019 for participant organizations in the NQMBC. The improvements were the greatest for those organizations that started in the lowest-performing percentiles. This is the goal of quality improvement in medicine—to decrease variation across the system and to bring those in the poorer-performing organizations toward the performance of better-performing institutions (Fig. 5). Based on these data, the NQMBC is providing breast centers with a tool that is contributing to improved quality of care over time. This is additional evidence that serial measurements and comparisons are powerful tools to encourage performance for institutions that are interested in quality.^13^

Interest in the timeliness of breast cancer care as a quality measure has only increased over the last 20 years.^2,14–16^ Numerous studies have demonstrated that time to treatment can impact survival for breast cancer patients with an understanding that delay worsens prognosis for patients with in situ and invasive carcinoma.^3,17^ This has been demonstrated even for the smallest of cancers, such as those identified on screening mammogram.^2^ Interdisciplinary breast centers are uniquely diverse, and breast cancer care is delivered by a spectrum of specialists, each with different offices, electronic medical records, and services provided. Care can be cohesive or fragmented, under one roof or many. Capturing quality in the breast cancer patient journey is a significant challenge, but the timing of each element contributes significantly to the overall time to treatment. Other than the time from surgical consult to the operating room, screening to diagnostic mammogram and diagnostic mammogram to biopsy taken together are the second largest contributors to pretreatment duration in the breast cancer patient experience. Anxiety associated with time delays can have lasting consequences for patients who are ultimately diagnosed with breast cancer, and those that receive benign results.^18–21^ Even for women who ultimately do not have a cancer diagnosis, anxiety, distress, and intrusive thoughts can persist for months to years after their diagnostic workup.^20,21^ To encourage patients to participate in consistent screening and decrease the emotional burdens of diagnostic workup, it is particularly important to minimize time to diagnosis. Centers in the NQMBC have significantly decreased the period of prediagnostic anxiety for patients, thereby improving patient care and the patient experience.

Without widely recognized benchmarks, it is valuable to know what timeliness of care looks like “in the real world.” How long are women waiting on average for these tests and results? For participants in the NQMBC, > 90% of centers averaged less than 13.5 days from screening to diagnostic imaging and > 75% of organizations averaged less than 10 days. Similarly, > 90% of institutions averaged < 13.7 days from diagnostic imaging to core needle biopsy and > 75% of organizations averaged < 9 days. The highest-performing institutions were able to narrow this time to 2–3 days, with some centers offering same-day biopsy for abnormal diagnostic imaging results. On average, patients might expect to have diagnostic mammograms and biopsies within 1 week. It is important to note that there are many elements that contribute to scheduling, and not all of them are under the control of the breast centers. We are mindful that we do not want these targets to become insurance company payment thresholds. The data have remained aggregate and anonymous intentionally. Often, standards dictated from averages across the country fail to encompass the many diverse and divergent elements that are involved in achieving them.

There have been other analyses that evaluated the time to treatment, but do not specifically address the prediagnosis time intervals. In evaluating time to mastectomy, barriers were uncovered at all levels from patient and disease factors to provider and systemic factors.^22^ Longer time to mastectomy for breast cancer patients has been demonstrated in African American patients. Non-Hispanic Black patients had 75–90th percentile times to surgery when compared with white women.^23,24^ Timeliness of treatment can also be impacted by financial and insurance factors.^25^ Patients with Medicaid insurance had increased time to mastectomy, and patients with greater financial concerns have longer delays in treatment.^26^ Primary care physician continuity has been implicated in timeliness to breast cancer care.^27^ Additionally, patients do not always choose the first available appointment due to personal scheduling conflicts or other concerns.^28^

NQMBC centers were polled by the NCBC in 2021 regarding methods to improve timeliness. Strategies used by centers to decrease time included adding open time slots, improving office organization, sharing timeliness data at breast conference, meeting with pathology to assess turnaround times, and optimizing the phone tree and front office.

Much has changed in the medical delivery system over the last 15 years, including the widespread adoption of the electronic medical record and the implementation of the Affordable Care Act. Increased access to screening, as was provided by the Affordable Care Act, has resulted in longer wait times to diagnostic procedures in some systems, while the electronic medical record has been shown to improve coordination and timeliness of patient care.^29,30^ Additionally, the incorporation of patient navigation has profoundly improved patient care. Whether they are housed in radiology, pathology, or in a patient care setting, navigators have made a significant contribution to reducing scheduling delays and patient anxiety.^31^

It is unclear what impact these individual and systemic factors have on the overall timeliness of care, but there are clearly regional differences in care delivery that remain statistically significant on multivariable analysis. The Midwest notably has the shortest time intervals, while the South Atlantic has the longest. It is not clear that these differences are attributable to patient, disease, or system-based characteristics or to population differences, but these findings pose questions about care delivery across the country. These data are specific to centers in the USA, but international data and comparisons could be helpful.

Surprisingly, screening volume and breast cancer volume did not significantly influence timeliness. It appears that organizations of different sizes have adjusted and adapted to take care of the volume they are used to treating. The way that screening and cancer volume impact imaging scheduling does not appear to result in advantages for one type of center over another. It should be noted here that COVID-19 has significantly impacted all aspects of cancer care, prolonging each phase from diagnosis to treatment.^32^ Screening tests ground to a dramatic and sudden halt. Currently, staffing issues are widespread, exacerbating and prolonging treatment delays across the country. There are several studies that are designed to answer some of these additional questions including the PROMPT study and a systemic meta-analysis.^33^ The NQMBC data presented here could serve as a target for a return to prepandemic levels of care delivery for breast centers dedicated to delivering quality care.

It is also important to note that participant organizations in the NQMBC report when and where they are able and that the NQMBC does not dictate how institutions should provide care. It does not say why timeliness is improving, only that it has improved. Additionally, as a volunteer program, centers were not required to submit for each 6-month period. While data are confidential and not reported elsewhere, centers may have preferentially submitted data when they had improved performance and conversely withheld data if their performance declined. It is also possible to have an incorrect data point from a center missed by random audit. However, the size of the database and the consistency of results minimize these concerns. It is also notable that NQMBC is not the only method for tracking timeliness in breast cancer care and other national databases give access to similar information for breast centers. The NQMBC tool is accessible for a variety of types of centers across the country, and the data are confidential but given in a context that allows for comparison to like institutions. Participants in the NQMBC are demonstrating this interest by registering and tracking their timeliness, which may skew the data towards high performance standards by looking only at the data from institutions that have put a premium on improving quality.

## Conclusions

The NQMBC is a time-tested tool for evaluating timeliness of breast cancer care across diverse breast cancer care delivery systems in the USA. Time from screening mammogram to diagnostic mammogram and from diagnostic mammogram to biopsy decreased from 2005 to 2019 (in the era before the COVID-19 pandemic). Successive improvement was seen in each 5-year segment compared with the prior 5 years. There are regional and care delivery differences in timeliness. Centers with the slowest times showed greatest improvement during the time interval, indicating the power of measurement and comparison for improving quality in breast cancer care.
